# Supplementary material for: The interactome of intact mitochondria by cross-linking mass spectrometry provides evidence for coexisting respiratory supercomplexes
Source: Mol Cell Proteomics. 2017 Dec 8;17(2):216–32. doi: 10.1074/mcp.RA117.000470 (PMC5795388; doi:10.1074/mcp.RA117.000470)
Supplement: Supplemental Data [file supp_RA117.000470_133922_0_supp_23978_fzffwf.zip › spectra_annotation/mito_spectra_annotation/150-1-12-1-14-1.pdf]

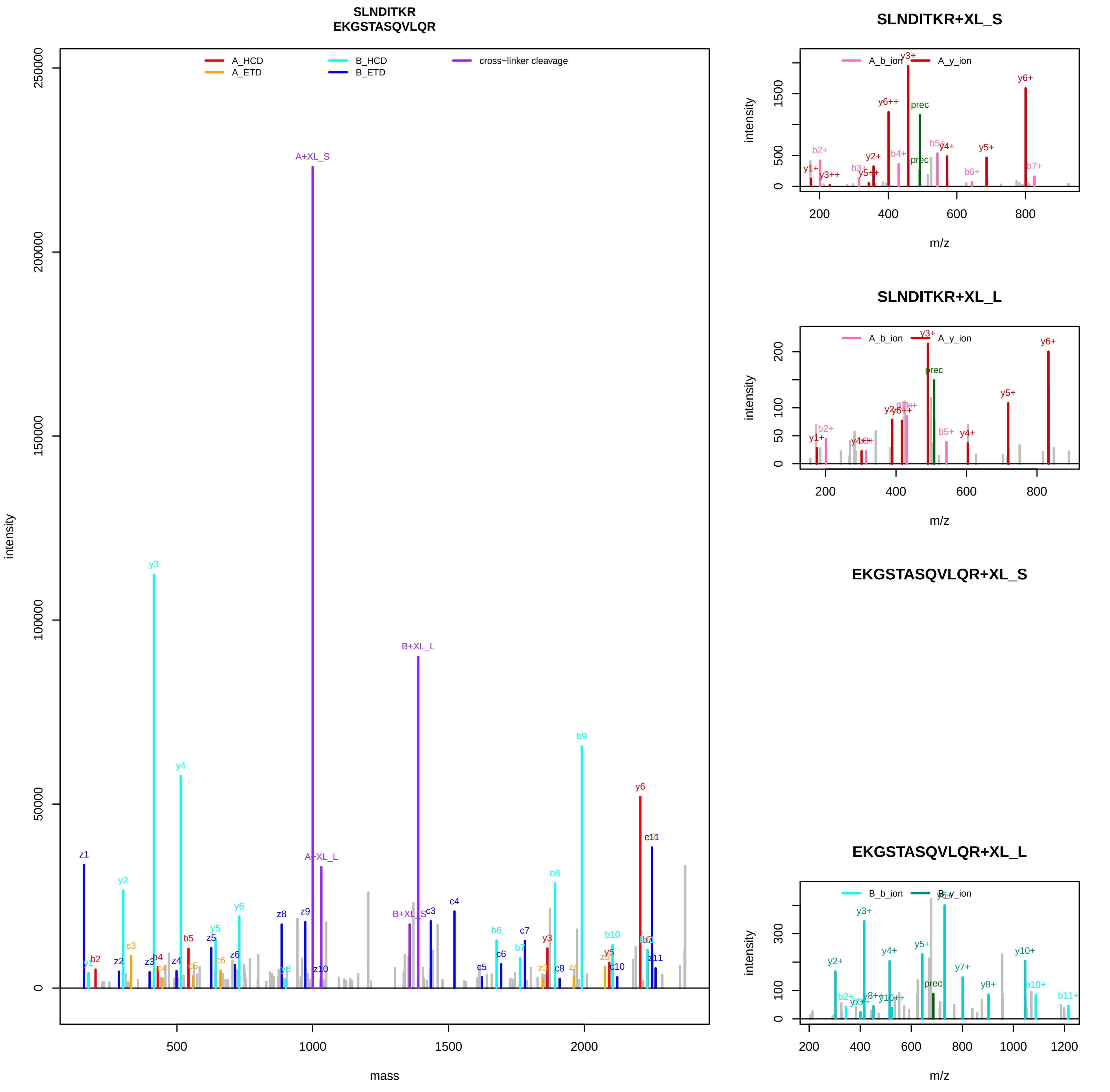
SLNDITKR
EKGSTASQVLQR
A_HCD
A_ETD
B_HCD
B_ETD
cross−linker cleavage
intensity
mass
0
50000
100000
150000
200000
250000
500
1000
1500
2000
A+XL_S
A+XL_L
B+XL_L
B+XL_S
z1
y1
b2
z2
y2
c3
z3
y3
b4
c4
z4
y4
b5
c5
z5
y5
c6
z6
y6
z8
y8
z9
z10
c3
c4
c5
b6
c6
b7
c7
y3
z3
c8
z4
b8
b9
z5
y5
b10
c10
y6
c11
z11
SLNDITKR+XL_S
A_b_ion
A_y_ion
intensity
m/z
0
500
1500
200
400
600
800
y1+
b2+
y3++
b3+
y5++
y2+
y6++
b4+
y3+
prec
b5+
y4+
b6+
y5+
y6+
b7+
SLNDITKR+XL_L
0
50
100
200
y1+
b2+
y4+
y2+
y6++
y3+
prec
b5+
y4+
y5+
y6+
EKGSTASQVLQR+XL_S
EKGSTASQVLQR+XL_L
B_b_ion
B_y_ion
0
100
300
1000
1200
y2+
b2+
y3+
y4+
y5+
prec
y7+
y8+
y10+
b10+
b11+
